# How to Measure Export via Bacterial Multidrug Resistance Efflux Pumps

**DOI:** 10.1128/mBio.00840-16

**Published:** 2016-07-05

**Authors:** Jessica M. A. Blair, Laura J. V. Piddock

**Affiliations:** Antimicrobials Research Group, Institute of Microbiology and Infection, College of Medical and Dental Sciences, The University of Birmingham, Birmingham, United Kingdom

## Abstract

Bacterial multidrug resistance (MDR) efflux pumps are an important mechanism of antibiotic resistance and are required for many pathogens to cause infection. They are also being harnessed to improve microbial biotechnological processes, including biofuel production. Therefore, scientists of many specialties must be able to accurately measure efflux activity. However, myriad methodologies have been described and the most appropriate method is not always clear. Within the scientific literature, many methods are misused or data arising are misinterpreted. The methods for measuring efflux activity can be split into two groups, (i) those that directly measure efflux and (ii) those that measure the intracellular accumulation of a substrate, which is then used to infer efflux activity. Here, we review the methods for measuring efflux and explore the most recent advances in this field, including single-cell or cell-free technologies and mass spectrometry, that are being used to provide more detailed information about efflux pump activity.

Multidrug resistance (MDR) efflux pumps are found in all bacterial species. They are protein complexes capable of transporting substrate molecules with various sizes and properties from the inside of the bacterial cell to the extracellular space. Bacterial efflux pumps are classified into five groups, the major facilitator superfamily (MFS), the small MDR (SMR) family, the multidrug and toxic compound extrusion (MATE) family, the ATP-binding cassette (ABC) family, and the resistance-nodulation-cell division (RND) family, which is the most clinically relevant in terms of antibiotic resistance. The MFS, SMR, MATE, and RND families all derive energy from the proton motive force, whereas the ABC transporters use direct hydrolysis of ATP to drive transport.

Bacterial efflux pumps have been best studied for their role in antibiotic resistance (1), but they are also fundamental to bacterial physiology and many are required for bacterial pathogens to cause infection (2) and to form biofilms (e.g., see references 3 and 4). In addition, export of substrates via efflux pumps is being harnessed to improve biotechnological processes (5–7). The myriad roles of these proteins mean that microbiologists of many specialties working on bacteria of all species research efflux pumps. Therefore, it is necessary to be able to quantify the activity of efflux pumps to understand their contribution to biological processes and to assess the validity of potential therapeutics such as efflux inhibitors.

When studying the efflux of antimicrobial substrates, it has been commonplace to use drug susceptibility measurements (such as the MIC) to reveal differences in drug efflux activity. The reason for using this method is that a bacterium with greater expression of an efflux pump will be less susceptible to various antimicrobials than its comparator with lower efflux pump expression. Two approaches have been typically taken. The first is a comparison of MICs obtained for isogenic laboratory strains in which a mutant has had a putative efflux gene inactivated or deleted. If the gene codes for an efflux pump that exports the drugs tested, the MICs are lower for the mutant, as higher concentrations of the drug are retained within the bacterium. In the presence of an influx inhibitor, the MICs are reduced for the parental strain but not for the mutant. The second is a comparison of MICs obtained in the presence and absence of inhibitors of drug efflux for a reference strain with those obtained for collections of clinical or veterinary isolates of bacteria. Those isolates with increased drug susceptibility in the presence of an efflux inhibitor are presumed to overexpress one or more efflux pumps. However, this approach has limited sensitivity and utility, as only large changes in efflux activity will be detected in this way. The fold changes in MICs also rarely correlate exactly with those obtained in experiments that determine efflux activity directly. Furthermore, the long time scale of susceptibility determination experiments (~18 to 24 h) means that subtle differences may be missed. Therefore, it is hard to be confident that the differences in the MICs are due to efflux; this is particularly true for studies comparing groups of isolates. Occasionally, disc susceptibility measurements are carried out instead of MIC determinations; as there are inherent difficulties in carrying out these experiments for certain species, e.g., *Pseudomonas aeruginosa*; data arising from this approach are even more unreliable. Therefore, direct ways of assessing efflux activity are necessary.

There are myriad methodologies described in the literature to measure the activity of efflux pumps in bacterial cells. Most use a molecule that is a substrate of the efflux pump under investigation and whose relative concentration can be easily detected, for example, by measuring fluorescence. Essentially, the methods can be split into two categories, (i) those that directly measure efflux, i.e., how much of a substrate is pumped out, and (ii) those that measure how much of a substrate molecule accumulates inside the cell, the level of which is then used to infer efflux activity indirectly.

Both types of method typically use dyes that have differential fluorescence when intra- or extracellular (Tables 1 and 2). Several substrates are used for these assays, including dyes that intercalate with DNA, but most commonly, dyes such as Hoechst H33342 or ethidium bromide are used. These fluoresce only when bound to DNA (8). Therefore, the level of fluorescence can be used to determine the relative intracellular concentration of the dye at any time point.

**TABLE 1:** Substrates commonly used to measure direct efflux

Substrate	Details	Advantage(s)	Disadvantage(s)	Reference(s)
Ethidium bromide	DNA-intercalating dye; fluoresces when bound to DNA; excitation wavelength, 530 nm; emission wavelength, 600 nm	Well-validated substrate of many efflux pumps such as the RND pump AcrB	Concentrates in cytoplasm, so efflux is slow because there must be a dissociation step and probably more than one efflux event; likely to underestimate efflux level	9, 41
Nile Red	Periplasmic; lipophilic dye that binds to membrane phospholipids; fluoresces weakly in aqueous solutions but strongly fluorescent in nonpolar environments such as the membrane; excitation wavelength, 552 nm; emission wavelength, 636 nm; assay uses stationary-phase cells	Periplasmic, so good for studying RND efflux pumps such as AcrB; long maximum emission wavelength (636 nm) means that there is less of a problem with interference when measuring competition with other substrates; better signal-to-noise ratio than ethidium bromide; efflux is more rapid than cytoplasmic dyes; can be used to test whether compounds are efflux substrates by measuring competition with Nile Red	Does not work well in nonfermenting bacteria such as *Pseudomonas* spp.; assay optimized for study of AcrAB-TolC	12
1,2′-Dinaphthylamine	Periplasmic; lipophilic dye that fluoresces weakly in aqueous solutions but strongly in nonpolar environments such as the membrane; more lipophilic than Nile Red, so ideal for studying RND efflux pumps because phenylalanines are important for substrate interaction in binding pocket; excitation wavelength, 370 nm; emission wavelength, 810 nm	Most sensitive; can be used to distinguish between efflux rates of AcrB proteins with SNPs;^a^ well retained in membrane while bacteria are in deenergized state; capable of emission in near-infrared region of spectrum, where cellular autofluorescence is low	Does not work well in nonfermenting bacteria such as *Pseudomonas* spp.	13
Doxorubicin	Fluoresces more extracellularly than intracellularly, so fluorescence increases upon efflux; excitation wavelength, 450 nm; emission wavelength, 600 nm		Very expensive	10, 42

a SNPs, single-nucleotide polymorphisms.

**TABLE 2:** Substrates commonly used to measure accumulation

Substrate(s)	Details	Advantages	Disadvantages	References
Hoechst H33342	DNA-intercalating dye; fluoresces when bound to DNA; excitation wavelength, 355 nm; emission wavelength, 460 nm	Easy and quick to use, cheap, and easily adapted for high throughput		20, 21
Ethidium bromide	DNA-intercalating dye; fluoresces when bound to DNA; excitation wavelength, 530 nm; emission wavelength, 600 nm	Easy and quick to use, cheap, and easily adapted for high throughput		8, 20
Fluoroquinolones (e.g., ciprofloxacin, norfloxacin)	Naturally fluorescent, but fluorescence is the same whether intracellular or extracellular; therefore, the assay is done differently; the drug is allowed to accumulate inside cells, cells are washed and then lysed, and the amount of drug in solution is measured by fluorescence and related to dry cell weight to estimate the amount of drug inside each cell	Arguably more clinically relevant than some dyes, as it measures accumulation of antibiotics	Single-time-point readings taken at timed intervals rather than real-time kinetics, but many time points can be taken	30, 33

## DIRECT MEASUREMENT OF EFFLUX

**FIG 1:**
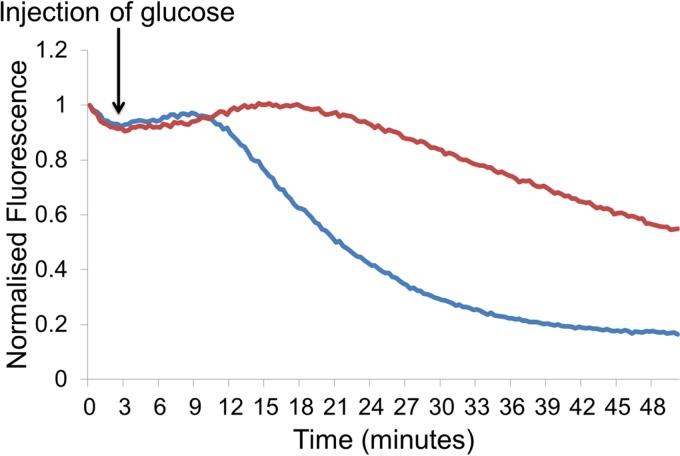
Direct measurement of ethidium bromide efflux (40) over time by *Salmonella* Typhimurium SL1344 (blue) and an isogenic Δ*acrB* mutant (red).

The principle of methods that measure efflux is that cells are preloaded with high concentrations of the substrate being measured, usually in the presence of an efflux inhibitor that inhibits the pump’s source of energy (e.g., carbonyl cyanide *m*-chlorophenylhydrazone, which dissipates the proton motive force, or orthovanadate, which inhibits transport via pumps that use ATP). This means that the substrate accumulates within the cell to a maximum level prior to the start of the assay. The cells are then washed to remove any extracellular substrate and inhibitor molecules remaining in the medium. The fluorescence of the cells is then measured. The initial fluorescence value is high because the cells have accumulated dye that they are unable to pump out. The cells are then reenergized, e.g., by the addition of glucose, allowing efflux to restart and the dye is effluxed out of the cells. Efflux is directly measured by recording the change in fluorescence over time as the dye is pumped out of the cells. The rate of efflux in different strains can be compared; for example, a strain of *Salmonella enterica* serovar Typhimurium lacking the AcrB efflux pump is unable to efflux ethidium bromide out of the cell as quickly as its isogenic wild-type parental strain (Fig. 1).

Ethidium bromide is a common substrate for these assays; it is a DNA-intercalating agent that fluoresces only when bound to DNA (8). Therefore, fluorescence is higher when intracellular than when extracellular, and this is used to measure the amount of accumulation (e.g., see references 9 to 10). Similar data can be obtained by using lipophilic dyes such as Nile Red and 1,2′-dinaphthylamine (12, 13). These dyes remain largely periplasmic and bind to membrane phospholipids, where they fluoresce more strongly than when in aqueous solution. Again, these dyes are more fluorescent when intracellular than when extracellular. Of these dyes, 1,2′-dinaphthylamine is currently the most sensitive and has the greatest signal-to-noise ratio, as its peak fluorescence is far from the region of the spectrum where cellular autofluorescence is detected. Nile Red and 1,2′-dinaphthylamine are particularly well suited to the measurement of efflux through RND efflux pumps. This is partly because of their periplasmic nature, as RND efflux pumps collect their substrates from the periplasmic space and outer leaflet of the inner membrane. This is an advantage over cytoplasmic dyes such as ethidium bromide, where efflux is slower; this is because the dye must first dissociate from the DNA. Efflux is probably a two-step process involving transport from the cytoplasm to the periplasm before export from the cell; this could lead to underestimation of the level of efflux. 1,2′-dinaphthylamine is more lipophilic than Nile Red and, because of the interaction with the phenylalanine-rich substrate binding pocket, is particularly good for studying RND efflux pumps (13). Another consideration when choosing a substrate is whether the results need to be interpreted in context with the protein structure. For this purpose, there are advantages to using a drug or dye for which the exact binding position within the pump is known. For example, doxorubicin has been cocrystallized with AcrB and therefore its binding location is known. This allows the effects of single-point mutations on the efflux level to be interpreted at the level of individual amino acid residues (e.g., see reference 10).

A major benefit of measuring efflux directly is that it can be used to obtain kinetic information about substrate export. More specifically, these assays can be used with a dye in combination with a molecule or drug thought to be a substrate or inhibitor of the efflux pump to determine whether there is competition for efflux. This approach has been used to infer whether a particular molecule is a substrate of a given efflux pump and even to which part of the efflux pump it binds (10, 14, 15). In addition, the sensitivity of this approach means that it can be used to screen for molecules that inhibit efflux and could be potential novel therapeutics (16–19).

## METHODS FOR MEASURING INTRACELLULAR ACCUMULATION OF AN EFFLUX PUMP SUBSTRATE

Many methods measure the accumulation of a substrate molecule inside the bacterial cell to infer the level of efflux (examples of commonly used substrates are shown in Table 2). The premise is that the lower the level of efflux, the higher the concentration of substrate accumulated within the bacterial cell. Historically, this was carried out by using radiolabeled substrates including antibiotics, and the level of radioactivity was used to determine the amount of accumulated substrate (22–25). Nowadays, it is far more common to use fluorescent substrates or dyes for this purpose. This is preferable, as the use of radioactivity is avoided. Fluorescence is not only easier to quantify, it allows easy scaling up of assays to a 96- or 384-well format, but it is also cheaper and easier to use and dispose of than radiochemicals. Common dyes include the DNA intercalation agents ethidium bromide and Hoechst H33342 (20, 21). These dyes fluoresce when bound to DNA and therefore have higher fluorescence when intracellular. A benefit of accumulation assays is that it is also possible to measure the accumulation of clinically relevant drugs such as the fluoroquinolones, which are naturally fluorescent (see discussion below).

**FIG 2:**
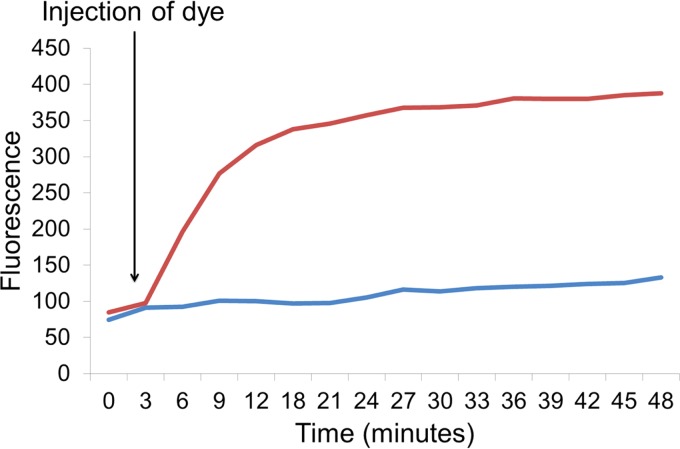
Measurement of Hoechst H33342 dye accumulation (data from reference 40) in *Salmonella* Typhimurium SL1344 (blue) and an isogenic Δ*acrB* mutant (red).

At the beginning of accumulation experiments, the bacterial cells have no dye or drug inside them; an initial fluorescence reading is taken, and dye is then added. The fluorescence increases over time as the dye accumulates inside the cells and reaches a steady state (Fig. 2). In an efflux-deficient strain (e.g., a strain from which an efflux pump gene has been deleted) the steady-state level of accumulation is higher than in an efflux-proficient strain (e.g., the wild type) because it is not able to pump the dye out of the cell as effectively. The relative fluorescence is used to compare levels of efflux between strains.

Measuring accumulation is most useful for comparing isogenic strains or highly related clinical isolates. For example, it has been used successfully in many species to measure changes in efflux/accumulation when genes coding for efflux pumps or their regulators are deleted or overexpressed in isogenic strains (e.g., see references 26 to 29). This method can also be adapted for many bacterial species, including nonfermenting bacteria that cannot metabolize glucose and therefore the real-time efflux assays described above are unsuitable (21, 26). While much of the discussion in this article has focused on pumps, such as the RND family, that use the proton motive force to power transport, measurements of drug accumulation can also be used to assess the activity of pumps that use alternative energy sources as long as the dyes or drugs selected for the assay are substrates of the pumps of interest. For example, accumulation assays have been used to assess the activity of members of the ABC family of pumps that use the hydrolysis of ATP to drive efflux (27–29).

However, accumulation is a less sensitive method than direct measurement of efflux and provides only indirect evidence of a change in efflux. This is because the amount of substrate that accumulates inside cells is also affected by other phenotypic attributes, including the rate of influx (the rate at which the dye enters the cell). Therefore, accumulation encompasses efflux activity and the contribution of influx to the overall level of substrate accumulation. This makes this method less well suited to assessing differences in efflux between nonisogenic strains, such as in collections of clinical isolates. This is because such isolates may have very different outer membrane permeabilities, causing different rates of substrate influx that will confound the quantification of efflux. For the same reason, data from accumulation assays cannot be used to infer efflux kinetics if the rate of substrate influx is not known. Therefore, in many cases, only the steady-state value is useful.

Accumulation methods can also be used to infer the level of efflux of substrates that do not have differential fluorescence intra- and extracellularly. These include many clinically relevant drugs, such as the fluoroquinolones or tetracyclines (e.g., see references 22, 28, 30, and 31). In this case, the substrate is allowed to accumulate intracellularly. Samples are taken, and temperature and pH are carefully controlled during the washing and lysing stages to prevent efflux restarting. Cells are then washed and lysed. The amount of molecule/drug present is then measured by fluorescence or absorbance, and the concentration within the bacterial cells is calculated as the number of milligrams per unit of dry weight of cells, the total cellular protein, or the number of cells present before lysis. Methods have been developed for use in various bacterial species and using many different drugs (e.g., see reference 30). An alternative method to measure accumulation of nonfluorescent substrates is to use radioactive analogs, and examples have been described for many drugs and bacterial species, including quinolones (32, 33, 34), tetracyclines (31), chloramphenicol (22), β-lactams (22), and rifampin (23).

One benefit of measuring accumulation is that it can be easily adapted for use with single-cell methods or those using more sophisticated detection techniques such as mass spectrometry (see below).

## RECENT DEVELOPMENTS IN EFFLUX MEASURMENT

Recently, methods have been described to assess efflux activity of bacteria with greater resolution, in single cells, in cell-free systems, and using a greater range of natural and synthetic substrates.

**FIG 3:**
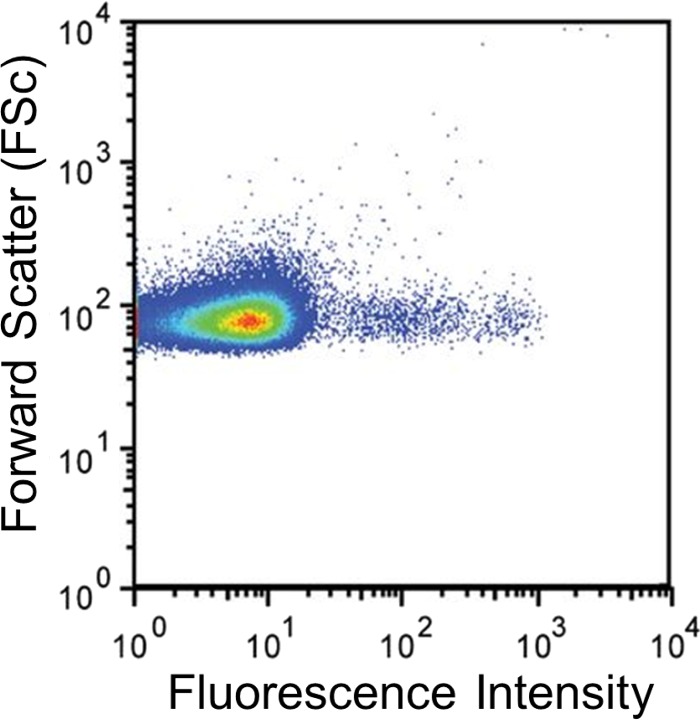
Ethidium bromide accumulation in an isogenic population of *S. enterica* showing variation in fluorescence throughout the population (data from reference 35).

The accumulation of fluorescent dye in single cells of a bacterial population can be measured by flow cytometry (e.g., see references 9 and 35). This technique allows the fluorescence from tens of thousands of individual cells from multiple populations to be measured. The benefit of this advancement is that the variation in efflux activity between cells in a population can be measured (Fig. 3) (9, 35). This has shown that the accumulation level, and therefore probably the efflux level, is not the same in all cells but varies within a population. This could be very important when considering the role of efflux in the emergence of resistance *in vivo* in the clinical setting. This variation in the level of dye accumulation across a population has also been described in eukaryotic cell populations (43).

One application of measuring efflux or accumulation is to screen for molecules that can inhibit efflux as potential future therapeutics. However, screening of natural products or complex mixtures for efflux inhibitory activity can be problematic, as they can contain molecules that cause optical interference when used with fluorescence-based methods. One method to circumvent this problem is to use quantitative mass spectrometry to measure the concentration to which a substrate has accumulated within cells by measuring the depletion of the substrate from spent liquid medium. For example, Brown and colleagues (36) have used high-performance liquid chromatography electrospray ionization-mass spectrometry to measure ethidium bromide uptake with the purpose of assessing efflux inhibition in *Staphylococcus aureus* by a crude plant extract and pure flavonoids. Many of the methods for measuring efflux or accumulation rely on the drugs being either fluorescent or radioactive so that their concentration can be measured. However, mass spectrometry-based methods of measuring drug accumulation are a major improvement because they can be used to measure the concentration of any substrate, including drugs (36).

All of the assays discussed thus far measure the efflux or accumulation of a substrate by efflux pumps in the context of the whole bacterial cell, where many other factors could influence the assay outcomes. Recently, an assay has been developed in which a tripartite RND efflux pump from *Pseudomonas aeruginosa* can be reconstituted by incorporating the inner and outer membrane components into separate proteoliposomes (37, 39). Assembly of the tripartite system and subsequent efflux by a specific pump can then be detected. Previously, only the inner membrane components had been incorporated into single proteoliposomes (e.g., see reference 38). This innovation has allowed conclusions about complex assembly. However, in the future, this method could be used to directly assess the impact of single-base-pair mutations on pump assembly with the major benefit of not being confounded or masked by other cellular functions. However, at present, the assay is only qualitative because of variability in the efficiency of reconstitution of the assembled pump and also in the reproducibility of the procedure.

## CONCLUSION

There are myriad methods available to assess efflux activity in bacterial cells, but they have differing levels of sensitivity and caution should be applied so that the method chosen is appropriate for the scientific question posed. When comparing isogenic mutants, accumulation of a fluorescent substrate is valid but this method is unsuitable for the comparison of phenotypically varied groups or collections of bacterial isolates because other physiological differences, such as differences in membrane permeability altering dye influx rates, could confound conclusions. Direct measurement of efflux is more sensitive than measurement of dye accumulation, provides more information, and is the only valid way to make kinetic measurements of efflux. Selection of the appropriate methodology is vital to draw sound conclusions about efflux activity.
